# First month of the epidemic caused by COVID-19 in Italy: current status and real-time outbreak development forecast

**DOI:** 10.1186/s41256-020-00170-3

**Published:** 2020-10-01

**Authors:** Rosario Megna

**Affiliations:** Institute of Biostructure and Bioimaging, National Council of Research, Naples, Italy

**Keywords:** COVID-19, SARS-CoV-2, Outbreak, Forecast model, Forecast in real-time, Epidemiology

## Abstract

**Background:**

The first outbreak of COVID-19 in Italy occurred during the second half of February 2020 in some areas in the North of the country. Due to the high contagiousness of the infection, further spread by asymptomatic people, Italy has become in a few weeks the country with the greatest number of infected people in the world. The large number of severe cases among infected people in Italy led to the hospitalization of thousands of patients, with a heavy burden on the National Health Service.

**Methods:**

We analyzed data provided daily by Italian Authorities for the period from 24 February 2020 to 30 March 2020. Considering such information, we developed a forecast model in real-time, based on the cumulative log-logistic distribution.

**Results:**

A total of 101,739 infected individuals were confirmed until 30 March 2020, of which 14,620 recovered or discharged, and 11,591 deaths. Until the same date patients quarantined at home were 43,752, whereas hospitalized patients were 31,776, of which 3981 in intensive care. The active cases (i.e. the number of patients not yet recovered until that date) were 75,528. The forecast model estimated a number of infected persons for Italy of 234,000 about, and a duration of the epidemic of approximately 4 months.

**Conclusions:**

One month after the first outbreaks there seemed to be the first signs of a decrease in the number of infections, showing that we could be now facing the descending phase of the epidemic. The forecast obtained thanks to our model could be used by decision-makers to implement coordinative and collaborative efforts in order to control the epidemic. The pandemic due to novel Coronavirus must be a warning for all countries worldwide, regarding a rapid and complete dissemination of information, surveillance, health organization, and cooperation among the states.

## Introduction

The outbreak of the novel Coronavirus called COVID-19 (or SARS-CoV-2), which had the first diagnosed cases at the end of December 2019 in the Hubei Province, China [1], is having a dramatic global evolution, and was recently classified as a pandemic by the World Health Organization (WHO) on 11 March 2020 [2]. The disease, which can be diagnosed through the use of a nasopharyngeal swab, under the most severe forms can lead to bilateral pneumonia [3] which can be lethal especially in elderly patients with comorbidities [4].

The initial detected outbreaks of COVID-19 in Italy occurred during the second half of February 2020 in some areas in the North of the country. First cases were diagnosed in southern Lombardy on February 21, on the border of the Veneto and Emilia Romagna regions [5–7]. On February 23, 11 municipalities were quarantined: nobody could enter and leave those territories (DL n. 6, 23 February 2020) [8]. Quickly other outbreaks occurred in the North of the country, requiring a wider extension of the area of limited human activities to various northern regions including Lombardy, Emilia Romagna, and Veneto (DPCM of 4 March 2020) [9]. Despite the drastic restrictions imposed by the Italian Government in those areas, several other outbreaks began in other areas of northern Italy, forcing the Authorities to extend the previously adopted restrictions to the entire national territory (DL of 9 March 2020, and DPCM of 22 March 2020) [10, 11]. Due to the high contagiousness of the infection, further spread by asymptomatic people [4, 12], in few weeks Italy became the country with the greatest number of infected people after China (confirmed cases greater than 80,000 from 26 March 2020). As a matter of fact, the virus spread globally so fast that currently the country with the highest number of cases is the US [13]. The large number of severe cases among infected people in Italy led to the hospitalization of thousands of patients [14, 15], with a heavy burden on the National Health Service [7]. In particular, the most affected regions were Lombardy and Emilia Romagna, with more than half of the total cases. It is reasonable to assume that the large spread of the novel Coronavirus in these regions was due to the development of the first outbreaks which caused a high number of people infected before the social distancing imposed by Government. Starting from 1 month after the initial outbreak in Italy, we reported the current status and proposed a forecast model in real-time to estimate its evolution in terms of epidemic duration and potential number of infected persons. This information could be applied in surveillance to inform clinicians and decision-makers to take coordinative and collaborative efforts to control the pandemic.

## Methods

Data on COVID-19 used in our analysis are daily updates from the Italian Ministry of Health managed by the Civil Protection Department [14, 15]. A report is released at 5:00 or 6:00 pm (CET), on the basis of information provided by National and Regional Local Authorities. The most relevant variable is the number of confirmed cases. The other derivate variables to be considered are the number of hospitalized patients (in intensive or non-intensive care), individuals quarantined at home, patients who recovered or were discharged, and of the number total deaths.

We analyzed data used in this study using the R software, version 3.6.3 (R Foundation for Statistical Computing, Vienna, Austria). Continuous variables were expressed as mean ± standard deviation and categorical variables as percentages, while differences between groups were evaluated by χ^2^ test for proportions.

We developed a forecast model in real-time, based on the cumulative log-logistic distribution [16, 17]. The equation used is the following:

1 $$ C(t)=\frac{N}{1+a\cdotp \mathit{\exp}\left(b\cdotp \mathit{\log}\left(t-{t}_0\right)\right)} $$

where *C*(*t*) is the cumulative incidence on day *t*, *N* is the cumulative incidence at the end of the epidemic, *a* and *b* are the parameters that govern the symmetry and the growth rate of the curve, and *t*_0_ is the initial day of the epidemic analysis (24 Feb 2020). In order to determine the three parameters of the curve, we developed an algorithm based on the maximum statistical significance of *a* and *b*, according to their *P*-values (smaller values of *P* indicate greater significance), and varying *N*. Nonlinear least squares (nls) function of R was used, normalizing *C*(*t*) → *C*(*t*, *N* = 1) ≡ *C*. The steps of the algorithm are the following:

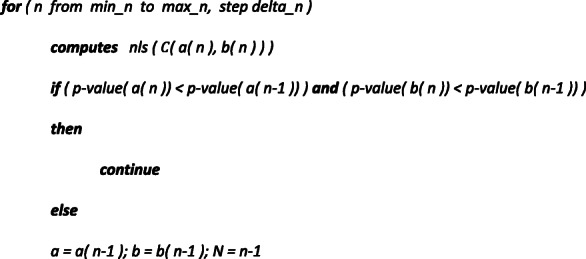



with min_n = 30,000, max_n = 300,000, and delta_n = 1000 for the National evaluation and min_n = 10,000, max_n = 100,000, and delta_n = 500 for the Regional evaluations (data used for the National model and computational R code are reported in the supplementary materials). At the end of the last cycle, the algorithm provides parameters for best-fit. In order assess the 95% confidence interval (CI) of the fit values, 1000 bootstrap resampling were computed, through the IPEC package of R. The date of the epidemic peak was computed by the maximum of the first derivative of *C*(*t*). Due to the asymptotic pattern of *C*(*t*), the date of the epidemic end was computed as the day with less than 100 new cases for the National evaluation, and less than 50 new cases for the Regional evaluations. The graphics were obtained using the ggplot2 package of R.

## Results

**Fig. 1:**
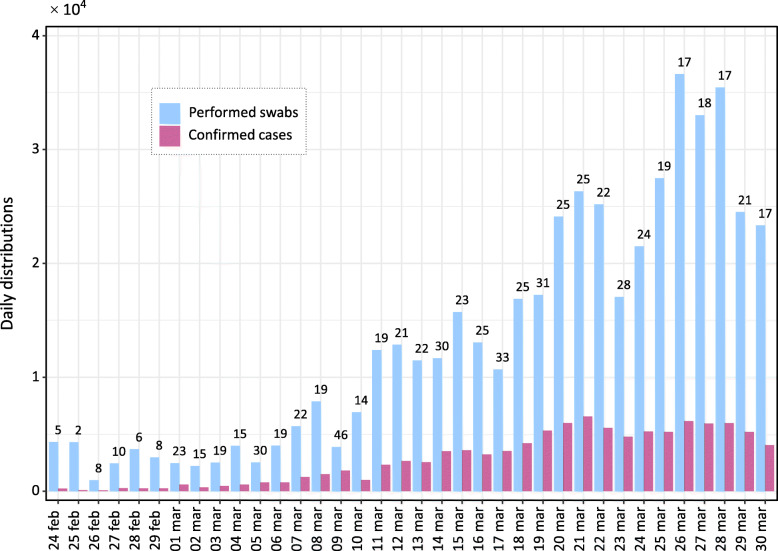
Daily distribution of the performed swabs and confirmed cases. The numbers are related to the ratio, in percentage, between confirmed cases and performed swabs

**Fig. 2:**
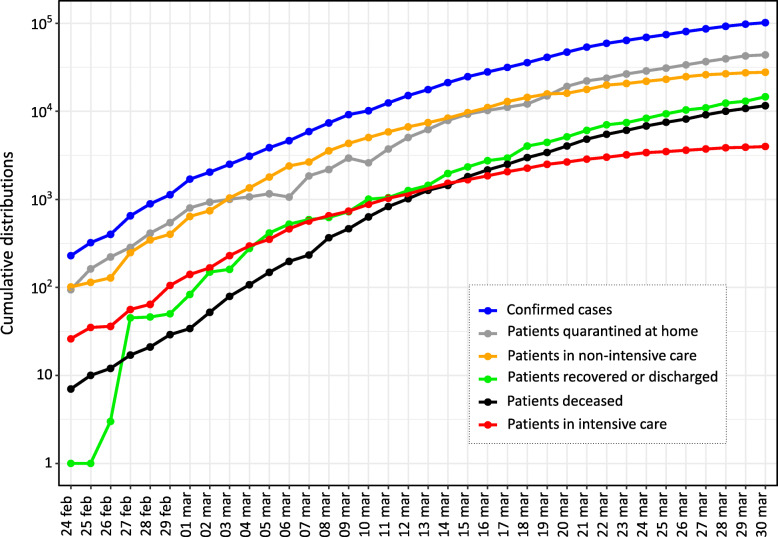
Cumulative distribution of the confirmed cases and patient categories

A total of 101,739 infected individuals were confirmed until 30 March 2020, of which 14,620 recovered or discharged, and 11,591 deaths. Until the same date, patients quarantined at home were 43,752, whereas hospitalized patients were 31,776, of which 3981 in intensive care. The active cases (i.e. the number of patients not yet recovered to the date) at the time were 75,528. Figure 1 shows the daily distribution of performed swabs and confirmed cases. The numbers over the bars are related to the ratio, in percentage, between the two variables (8.7% ± 7.7%). Until 30 March 2020, the total number of performed swabs was 477,359. Figure 2 shows the cumulative distributions of the confirmed cases and patient categories. During the first month of the epidemic, the confirmed cases increased of almost three orders of magnitude, the trends of patients quarantined at home and in non-intensive care remained very similar among them, whereas from mid-March the number of deceased individuals (greater than 1000 per day) exceeded the patients in intensive care. In Table 1 we reported the number of confirmed cases and the patient categories at a National and Regional level (Lombardy and Emilia Romagna). The confirmed cases to date were greater than 100,000, the hospitalized patients were greater than 40,000 as well the persons quarantined at home, while the number of deceased patients was significantly greater than the Chinese [13, 18] (*P* < 0.001).

**Table 1:** Confirmed cases and patient categories updated to 30 March 2020

	Italy	Lombardy	Emilia Romagna
Confirmed cases	101,739	42,161	13,531
Quarantined at home	43,752 (43.0)	11,861 (28.1)	6636 (49.0)
Hospitalized in non-intensive care	27,795 (27.3)	11,815 (28.0)	3779 (27.9)
Recovered or discharged	14,620 (14.4)	10,337 (24.5)	1227 (9.1)
Deceased	11,591 (11.4)	6818 (16.2)	1538 (11.4)
Hospitalized in intensive care	3981 (3.9)	1330 (3.2)	351 (2.6)

Between brackets we report the ratio, in percentage, between each patient category and the confirmed cases

**Fig. 3:**
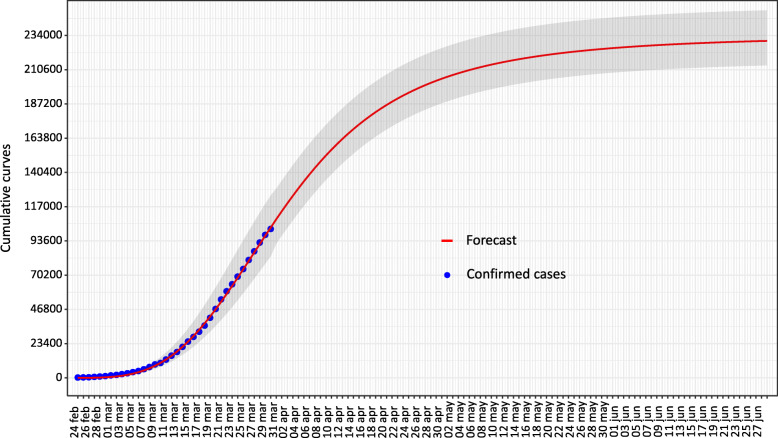
Cumulative curve obtained by the forecasted model of the epidemic in Italy in real-time. Predictions are represented by the red line, with the gray area to indicate 95% CI. The blue points represent the confirmed cases

In Table 2 we summarized the forecasted method results for at a National and Regional level (Lombardy and Emilia Romagna). The model predicted a number of the infected persons of 230,000 in Italy, of which 90,500 in Lombardy, and 44,000 in Emilia Romagna. The duration of the epidemic was estimated to be approximately of 4 months. Figure 3 shows the cumulative log-logistic curve obtained by the forecast model in real-time for the National overview.

**Table 2:** Best-fit parameters and relevant dates obtained by the forecasted model for the epidemic caused by COVID-19 in Italy

	Italy	Lombardy	Emilia Romagna
Best-fit parameters			
A	3.67·10^5^ *	7.29·10^4^ *	2.71·10^5^ *
B	−3.51 *	−3.10 *	− 3.28 *
N^a^	234,000 (214,000-251,000)	90,500 (82,500-97,500)	44,000 (38,500-51,000)
Relevant dates^b^			
Peak	27 March (33)	24 March (30)	1 April (38)
End	28 June (126)	27 June (125)	19 June (117)

* *P* < 10^−5^

^a^Between brackets we report 95% CI

^b^Between brackets we report the number of days since the outbreak started

## Discussion

One month after the outbreak in Italy the situation remains complicated. Despite the high number of performed swabs compared to the confirmed cases, the epidemic kept on growing at very speed. COVID-19 proved to have a high capacity for infection, probably reinforced by asymptomatic people, representing a real danger for elderly and fragile individuals. In particular, the disease is showing to be lethal for the elderly (95.2% in patients aged ≥60) and men (70.8%) [18]. On the date we finalized this article (30 March 2020), the trend of daily distribution of confirmed cases seems to show an initial decline of the growth of the epidemic. However, the total number of confirmed cases already exceeds those that occurred in China at this point in time. In addition to this, many deaths were not recorded as due to COVID-19 (there was no possibility of checking if the deceased had been infected) and therefore not recorded as such [19, 20]. The data related to the patient categories give us an estimate of the epidemic in terms of cases that can be treated at home, those who need hospitalization, and the mortality. The implications in terms of public health, workload in hospitals, and economic damage were worrisome. The hospital beds, in particular in intensive care, were saturated in several areas of Northern Italy [21–23], while the lockdown imposed from the Government to limit the infections has stopped almost all commercial activities in the whole country. In general, through the Italian epidemiological findings, countries with similar characteristics to those present in Italy (demographic characteristics of the population, health structures, etc.), should take earlier restrictive measures and arrange the necessary treatments for potential patients.

The forecast model in real-time indicates a total number of national cases greater than 230,000 patients, with a figure of approximately 90,000 in Lombardy only. In addition, the model estimates the duration of the epidemic in approximately 4 months. Since the theoretical cumulative curve has an asymptotic pattern (i.e. the maximum value is achieved for the t time towards infinite), considering 100 new cases in a day as the end of the outbreak is a convention. Therefore, if we considered 50 new cases in a day instead of 100, then the overall timeline estimated for the epidemic to come to an end would increase by approximately 21 days. Instead, if we considered 200 new cases, then the timeline would decrease by approximately by 19 days.

Moreover, several factors could affect the total number of cases and the duration of the epidemic. For example, a contribution to the spread of outbreaks in southern Italy was caused by the movement of students and workers from Northern to Southern Italy following the first governmental restrictions. On the other hand, more stringent restrictions imposed later on by the Government could lower the expected number of total cases and reduce the number of days towards the end of the epidemic. On this specific topic, a previous study on SARS-CoV-2 in China found a nonlinear and chaotic behavior of the virus, which emerged gradually but was highly responsive to massive interventions [24]. Another important factor is related to possible mutations of the novel Coronavirus [25], which could have a positive or negative outcome on the trend of the pandemic.

Considering what happened in Italy, other EU countries should adopt agreed measures regarding health and economic aids, and also regulate uniformly the movement of people among the member States, to avoid a new spread of the SARS-CoV-2. As a matter of fact, the pandemic due to novel Coronavirus is the most widespread in the globalization era, and the lesson about what is happening must be a warning for all countries worldwide. A rapid and thorough dissemination of information, surveillance, optimization of health systems, and cooperation among states is needed in order to reduce contagion and economic damages.

It is also necessary to consider the intrinsic limitations of this study. First of all, data was not always updated on a daily basis by each Regional Authority (an extract of the warnings list provided from Civil Protection is reported in the supplementary materials, Table SM 1). This limitation can have effects on the trend of the epidemiological curve, therefore on the fit of the data. Another limitation is represented of the reported cumulative counts, that are known be under-reported, especially at the beginning of the pandemic due to public awareness. If the counts are under-reported in the beginning of pandemic, all reported accumulated counts would be all under-estimated. We also have to consider that the number of infected people is underestimated, since there are many undetected asymptomatic individuals. These individuals can accidentally infect several other persons contributing to the spread of the epidemic. More specifically, in Italy 5.9% of individuals who had a check through a swab were diagnosed as asymptomatic and 12.9% were considered people with non-specific symptoms [18]. Such percentages could be underestimated, since the majority of the population did not take a swab. Finally, the factors that determine the trend of the epidemic curve could change without respecting the pattern of the forecasted model. As a matter of fact, the infected population growth is exponential at the beginning (as also verified in [26]), but tends to flatten towards the end due to saturation. Likewise, the tail-end of the log-logistic curve will be governed by the quarantined population and the consequent social distancing. Ultimately, the epidemic could end either through immunization of individuals affected (herd immunity) or thanks to the extinction of the virus. Since the overall number of infected citizens did not reach the majority of the population (approximately 60 million), the end of the epidemic is expected to occur with the extinction of the virus.

## Conclusions

The epidemic caused by COVID-19 in Italy is having a dramatic evolution in terms of confirmed cases, hospitalized and deceased patients. After 1 month since the first outbreaks, first signs of a decrease in the number of infections became apparent, showing the possibility of a descending phase of the epidemic. The model presented in this article fits well with the data, therefore it is expected to be reliable in predicting the evolution of the epidemic within the limits discussed. The forecast could be applied by decision-makers to take coordinative and collaborative efforts to control the epidemic. The pandemic due to novel Coronavirus is characterized by a fast spread worldwide, with dramatic repercussions on the health of the population and the economy.

## Supplementary information

**Additional file 1.**

**Additional file 2.**

## Data Availability

The data that support the findings of this study are available from http://opendatadpc.maps.arcgis.com/apps/opsdashboard/index.html#/b0c68bce2cce478eaac82fe38d4138b1
