# Supplementary material for: First month of the epidemic caused by COVID-19 in Italy: current status and real-time outbreak development forecast
Source: Glob Health Res Policy. 2020 Oct 1;5:43. doi: 10.1186/s41256-020-00170-3 (PMC7544517; doi:10.1186/s41256-020-00170-3)
Supplement: Supplementary file 1 — Additional file 1. [file 41256_2020_170_MOESM1_ESM.docx]

**First month of the epidemic caused by COVID-19 in Italy: current status and real-time outbreak development forecast**

Rosario Megna^*^, PhD

Institute of Biostructure and Bioimaging, National Council of Research, Naples, Italy

**Supplementary materials**

The supplementary materials consisting in:

- The file of data (*data.csv*) used for our analysis and forecasted model building. The variables related to the number of days, daily performed swabs, and ratio between daily performed swabs and daily confirmed cases were obtained by simple software routines in R.
- Computational R code for the forecasted model.
- Extract of the warnings list provided from Civil Protection, related to the partial or missing data communication in the understudy period.

**Computational R code for the forecasted model**

### Loading data ###

library(readr)

data <- read_csv("data.csv") # data.csv file is a part of the supplementary materials

data <- as.data.frame(data)

### Setting for model-building ###

nv = seq(from = 30000, to = 300000, by = 1000)

f <- function(x, a, b){1/(1+a*exp(b*log(x)))}

ls <- list()

### Cycle for evaluating best-fit parameters ###

for(k in 1:length(nv)){

fit <- nls(data$confirmed_cases/nv[k] ~ f(data$n_days, a, b), data=data, start=list(a=1, b=1),

control=c(maxiter = 200, tol = 1e-05, minFactor = 1/1024))

ls[[k]] <- summary(fit)

if(k > 1){

if(ls[[k]]$coefficients[7] < ls[[k-1]]$coefficients[7] &

ls[[k]]$coefficients[8] < ls[[k-1]]$coefficients[8]){

next

}

else{

print(nv[k-1]) # value of the “N” parameter

print(ls[[k-1]]) # value of the “a” and “b” parameters

break

}

}

}

**Table SM 1** Extract of the warnings list provided from Civil Protection, related to the partial or missing data communication in the understudy period [11]

| Date | Dataset | region | province | type_warning | warning | notes |
| --- | --- | --- | --- | --- | --- | --- |
| 2020-03-07T17:00:00 | dati-province | Lombardia | Brescia | partial data | partial communication of data | 300 positive cases |
| 2020-03-07T17:00:00 | dati-regioni | Lombardia | Brescia | partial data | partial communication of data | 300 positive cases |
| 2020-03-07T17:00:00 | dati-andamento-nazionale | Lombardia | Brescia | partial data | partial communication of data | 300 positive cases |
| 2020-03-10T17:00:00 | dati-regioni | Lombardia |  | partial data | partial communication of data |  |
| 2020-03-10T17:00:00 | dati-andamento-nazionale | Lombardia |  | partial data | partial communication of data |  |
| 2020-03-11T17:00:00 | dati-regioni | Abruzzo |  | no data | no data has been sent |  |
| 2020-03-11T17:00:00 | dati-andamento-nazionale | Abruzzo |  | no data | no data has been sent |  |
| 2020-03-16T17:00:00 | dati-regioni | P.A. Trento |  | no data | no data has been sent |  |
| 2020-03-16T17:00:00 | dati-andamento-nazionale | P.A. Trento |  | no data | no data has been sent |  |
| 2020-03-16T17:00:00 | dati-regioni | Puglia |  | no data | no data has been sent |  |
| 2020-03-16T17:00:00 | dati-andamento-nazionale | Puglia |  | no data | no data has been sent |  |
| 2020-03-17T17:00:00 | dati-province | Emilia-Romagna | Rimini | no data | no data has been sent |  |
| 2020-03-17T17:00:00 | dati-regioni | Emilia-Romagna | Rimini | no data | no data has been sent |  |
| 2020-03-17T17:00:00 | dati-andamento-nazionale | Emilia-Romagna | Rimini | no data | no data has been sent |  |
| 2020-03-18T17:00:00 | dati-province | Emilia-Romagna | Parma | no data | no data has been sent |  |
| 2020-03-18T17:00:00 | dati-regioni | Emilia-Romagna | Parma | no data | no data has been sent |  |
| 2020-03-18T17:00:00 | dati-andamento-nazionale | Emilia-Romagna | Parma | no data | no data has been sent |  |
| 2020-03-18T17:00:00 | dati-regioni | Campania |  | no data | no data has been sent |  |
| 2020-03-18T17:00:00 | dati-andamento-nazionale | Campania |  | no data | no data has been sent |  |
| 2020-03-26T17:00:00 | dati-regioni | Piemonte |  | partial data | partial communication of data | -50 death - late communication |
| 2020-03-26T17:00:00 | dati-andamento-nazionale | Piemonte |  | partial data | partial communication of data | -50 - late communication |
| 2020-03-29T17:00:00 | dati-regioni | Emilia-Romagna |  | partial data | partial communication of data | tests data not updated |
